# Food security status and cardiometabolic health by sex/gender and race/ethnicity among adults in the United States

**DOI:** 10.1186/s12889-024-18655-y

**Published:** 2024-05-03

**Authors:** Jamie A. Murkey, Symielle A. Gaston, Dana M. Alhasan, Christopher W. Payne, W. Braxton Jackson, Chandra L. Jackson

**Affiliations:** 1 Epidemiology Branch, National Institute of Environmental Health Sciences, National Institutes of Health, Department of Health and Human Services, Research Triangle Park, Durham, NC 27709 USA; 2 Social & Scientific Systems, Inc., a DLH Holdings Company, Durham, NC 27703 USA; 3 Intramural Program, National Institute on Minority Health and Health Disparities, National Institutes of Health, Department of Health and Human Services, Bethesda, MD 20892 USA

**Keywords:** Food insecurity, Food assistance, Ideal cardiovascular health, Cardiovascular disease, Health inequities, Social determinants of health

## Abstract

**Background:**

Minoritized racial/ethnic groups and women in the United States (US) are disproportionately burdened by food insecurity, which likely contributes to disparities in cardiovascular health (CVH). Disparities are projected to widen due to the worsening climate crisis that is straining the agricultural system including food supplies. Nonetheless, studies have not investigated the relationship between food security status and ‘ideal’ CVH in a large, nationally-representative and racially/ethnically diverse US sample.

**Methods and results:**

We investigated household food security status in relation to ‘ideal’ CVH among US adults (*N* = 157,001) using 2014–2018/2020 National Health Interview Survey data. Food security status was defined as very low, low, marginal, or high. A summed score of 4 health behaviors and 3 clinical factors totaling 7 different measures was dichotomized (yes/no) to assess modified ‘ideal’ CVH (mICVH). Using Poisson regression with robust variance, we estimated prevalence ratios (PRs) and 95% CIs of mICVH by household food security status. We stratified models by sex/gender and race/ethnicity. Very low food security prevalence was higher among non-Hispanic (NH)-Black (8.0%) compared to Hispanic/Latinx (5.1%), NH-White (3.1%) and NH-Asian (1.7%) adults. The association between very low versus high food security and mICVH was stronger among women (PR = 0.23 [95% CI: 0.17–0.31]) than men (PR = 0.48 [95% CI: 0.35–0.66]). Compared to NH-White adults with high food security, racially/ethnically minoritized groups with very low to high food security were generally less likely (range: [PR_very low_ = 0.25[95% CI: 0.14–0.44] – [PR_high_ = 0.88 [95% CI: 0.79–0.97]) to meet mICVH criteria.

**Conclusions:**

Food insecurity was associated with lower mICVH prevalence and racially/ethnically minoritized groups were disproportionately burdened.

**Supplementary Information:**

The online version contains supplementary material available at 10.1186/s12889-024-18655-y.

## Introduction

Food insecurity disproportionately burdens minoritized racial/ethnic groups and women in the United States (US) [1] and likely contributes to observed disparities in cardiovascular health (CVH) [2]. Although food is essential for life, food insecurity, defined as a lack of access to nutritious substances due to financial or resource constraints, is a major challenge in the US [1]. For instance, the prevalence of food insecurity was 10.5% among the overall population of US households in 2020 and was substantially higher among racially/ethnically minoritized households (e.g., 21.7% for Non-Hispanic (NH) Black) [3]. Additionally, households headed by NH-Black or Hispanic adults, women, or a single parent are the most likely to experience food insecurity [1, 4–6].

Inequities in food insecurity can be mapped to a variety of environmental and social factors. For instance, as climate change intensifies with more frequent and widespread natural disasters that strain agricultural systems and ultimately regional food supplies, existing inequities in food security status are projected to worsen [7, 8]. Diets rich in plant-based foods and with fewer animal products have been found to confer both improved health and environmental benefits, but access to these diets are inequitably distributed [9]. Furthermore, increasing effort has focused on understanding the contribution of social determinants of health—the conditions in which individuals live, grow, work, play and age—which are more downstream or proximal factors influencing health [10–12]. However, social determinants in the US have largely been shaped by more upstream factors such as globalization, structural racism, as well as federal, state, and local policies [11]. For example, historical and current supermarket, redlining practices in the US have hindered opportunities for social mobility and contributed to food insecurity through, for instance, a lack of community investments leading to ‘food deserts’ (areas devoid of healthy food options) as well as food swamps (areas concentrated with energy-dense, low-nutrient foods) which tend to be largely clustered in and around low-income neighborhoods as well as neighborhoods primarily comprised of racially/ethnically minoritized groups [13–17]. Notably, the term ‘food deserts’ has been met with much criticism among scholars and activists, as it inaccurately depicts the context of structural racist practices antecedent to the lack of healthy food options in some areas [14, 18]. Using the term food apartheid—which refers to the racist structures and practices that led to inequitable food environments—has instead been strongly recommended as a replacement for ‘food deserts’ [19, 20].

Food insecurity can affect health behaviors, such as diet, which makes achieving a healthy and balanced diet more difficult [21]. In fact, food insecurity may contribute to both malnutrition and obesity risk, directly exacerbating CVH disparities, particularly among households headed by women within racially/ethnically minoritized groups [1, 4, 5, 21]. The aforementioned food apartheid may also promote CVH disparities. For example, heads of households encountering financial hardship may decide to purchase high-energy, low-nutrient foods as way to feed their family within their budgetary constraints, even when low-energy, nutrient-dense options are present but unaffordable. Food insecurity has also been associated with poor sleep (via stress related to worrying about food, hunger, and the consumption of energy-dense, low nutrient foods) which is an important component of CVH [22–25].

Achieving and maintaining ‘ideal’ CVH (ICVH) — a key metric for ascertaining cardiovascular disease (CVD) risk factors using the American Heart Association’s (AHA) *Life’s Essential 8* — is based on eight key measures that include smoking status, body mass index (BMI), physical activity, diet, total cholesterol, blood pressure, fasting glucose, and sleep duration (an understudied but more recently recognized CVD risk factor) [25, 26]. Prior literature suggests that a higher number of ICVH metrics correlates with lower cumulative CVD incidence, as well as both all-cause and CVD-mortality risk [27, 28]. Prior studies have observed that women are more likely to be food insecure and have lower ICVH prevalence than men [1, 4, 5, 29, 30]. Additionally, while the prevalence of meeting ≥ 5 ICVH metrics is 45% among US adults, one prior study observed that ICVH prevalence was three times higher among NH-White compared to NH-Black and Hispanic/Latinx adults [26, 31, 32]. Since food insecurity is higher and ICVH prevalence is lower among racially/ethnically minoritized groups, studies assessing the relationship between the two are needed. Moreover, as more extreme weather events continually reoccur, disparate impacts in communities primarily consisting of racially/ethnically minoritized groups are expected to exacerbate CVH risk driven by social determinants of health inequities [33].

While it is known that food insecurity contributes to racial/ethnic disparities in health, few studies have determined the relationship between food security status and ICVH prevalence (especially using the updated metric including sleep duration) in a racially/ethnically diverse, nationally representative sample of US adults [1, 6, 21, 23, 24]. Further, fewer studies have employed an intersectional framework—the way in which the interconnectedness of race, gender, socioeconomic status, and other systems of power shape oppression and privilege—to investigate food insecurity in relation to ICVH inequities, an important consideration for conducting health disparities research [34]. Therefore, we addressed these important gaps in the literature by assessing food security status in relation to modified ‘ideal’ CVH (mICVH) prevalence among US adults. We hypothesized that individuals with ‘very low’ and ‘low’ as well as ‘marginal’ vs. ‘high’ food security status will have the lowest prevalence of mICVH and that lower levels of food security status would be associated with lower mICVH prevalence. Additionally, given findings from prior literature, we hypothesized that associations between food security status and mICVH prevalence would be stronger among women compared to men. Lastly, considering that food insecurity is higher and mICVH prevalence is lower among racially/ethnically minoritized groups compared to NH-White adults, we hypothesized that the association between food security status and mICVH prevalence would be the strongest among minoritized racial/ethnic groups.

## Methods

### Study population

The National Health Interview Survey (NHIS) is a nationally representative study that uses three-stage cluster probability sampling to administer interviews to individuals residing in non-institutionalized households in the United States. Study design and recruitment details for the NHIS study have been previously described [35]. We used 2014–2018 and 2020 cross-sectional NHIS data. NHIS participant data from 2019 were excluded from our study, as data on sleep duration—a component of the AHA’s ‘ideal’ CVH metric—were not collected during the 2019 survey year. All participants in the NHIS study provided written informed consent. Additionally, the use of non-identifiable, publicly available NHIS data was deemed exempt from approval by the National Institute of Environmental Health Sciences Institutional Review Board. The response rate among participants in our study was 49% (range: 58.8% (2014) – 45.2% (2020)).

Participants were eligible for inclusion in our study if they were ≥ 18 years, NH-Asian, Hispanic/Latinx, NH-Black, or NH-White. Additional racial/ethnic groups were not included due to small sample sizes. These criteria resulted in a sample of 182,056 adults. Further, we excluded NHIS participants if they were missing data on the exposure, outcome, and potential confounders: food security status, mICVH metrics (smoking status, BMI, physical activity, sleep duration, hypertension, pre-diabetes/type 2 diabetes, and dyslipidemia), age, sex/gender, race/ethnicity, annual household income, educational attainment, marital status, or alcohol consumption (*n* = 25,055). The exclusions resulted in a final analytic sample of 157,001 participants (Supplemental Figure 1).

### Exposure assessment: household food security status

Food security status data were collected using the U.S. Department of Agriculture’s (USDA) U.S. Household Food Security Survey Module, a 10-item screener (derived from the full 18-item U.S. Household Food Security Survey Module) routinely used to monitor food security [36]. The full 18-item scale has been shown to have good reliability (Cronbach α = 0.81 (for households with children) and 0.74 (for all households)) [37]. Participants were asked about household availability and consumption of food in the past 30 days. For example, participants were asked how often (often true; sometimes true; never true; or don’t know) the following happened in the past 30 days: “we worried whether our food would run out before we got money to buy more”; “we couldn’t afford to eat balanced meals”; “we were hungry but did not eat because there was not enough money”. Additionally, participants were asked whether or not (yes versus no) the following occurred during the past 30 days: “did you ever cut the size of meals or skip meals because there wasn’t enough money for food?”; “did you ever eat less than you felt you should because there wasn’t enough money for food?”; “did any of your family not eat for a whole day because there wasn’t enough money for food?”. The remaining questions have been summarized in Supplemental Table 1. Participants responding to an item affirmatively as “yes”, “often true”, or “sometimes true” were counted as 1 [38]. Responses were summed (0–10) and categorized as very low (6–10), low (3–5), marginal (1–2), and high (0) food security [38].

### Outcome assessment: modified ideal cardiovascular health

We constructed a mICVH metric using a summed score of 4 health behaviors and 3 clinical factors, which totaled 7 different measures [26]. The metrics included: 1) smoking (never smoked/quit smoking > 12 months prior to study enrollment); 2) BMI (≥ 18.5 kg/m^2^ and < 25 kg/m^2^); 3) physical activity (≥ 150–300 min/week moderate or ≥ 75–150 min/week vigorous [39]); 4) sleep duration (7 to 9 h of sleep nightly); and no prior diagnosis of 5) dyslipidemia, 6) hypertension, or 7) prediabetes/diabetes. Participants who reported “yes” to all of these measures were considered to have mICVH, and participants who reported “no” to any of these measures we not considered to have mICVH. This metric is considered modified because data on diet were not collected in the NHIS.

### Potential confounders

Potential sociodemographic and lifestyle confounders were selected *a priori*. Sociodemographic variables included age (18–30, 31–49, or ≥ 50 years); sex/gender (men or women); annual household income (< $35,000; $35,000-$74,999; ≥ $75,000); educational attainment (< high school, high school graduate, some college, or ≥ college); geographic region (Northeast, Midwest, South, or West); marital status (married/living with partner or cohabitating, divorced/widowed/separated, or single/no live-in partner); and survey year [5, 40]. Race/ethnicity (NH-Asian, NH-Black, Hispanic/Latinx, or NH-White) was also considered as a confounder for overall models. Lastly, we considered alcohol consumption (current, [heavy], current [≤ moderate], former or lifetime abstainer) as a lifestyle variable [40].

### Potential modifiers

Based on prior literature revealing more social vulnerability to food insecurity as well as CVD among both minoritized racial/ethnic groups and women, we investigated potential effect modification by sex/gender (men or women) and race/ethnicity (NH-Asian, Hispanic/Latinx, NH-Black or NH-White) [1, 4–6].

### Statistical analyses

We reported mean ± standard error for age and age-standardized (based on the 2010 US Census population) along with weighted percentages (to account for the complex survey design) for sociodemographic, lifestyle, health behavior, and clinical factors in the overall population and by food security status category. Poisson regression with robust variance was used to estimate prevalence ratios (PR’s) and 95% confidence intervals (CI’s) for associations between food security status and mICVH, adjusting for sociodemographic and lifestyle confounders. High food security status was the reference group to make comparisons to very low, low, and marginal food security status. We investigated potential differences in the association between food security status and mICVH by and sex/gender and race/ethnicity through stratification and by including multiplicative interaction terms in the models and testing their significance with a Wald test of interaction terms. In a separate analysis, we used NH-White adults with high food security as the reference group to compare racially/ethnically minoritized groups with very low, low, marginal, and high food security and mICVH. We used a two-sided alpha level of 0.05 to determine statistical significance in all analyses. All analyses were conducted using Stata version 15 (StataCorp LLC, College Station, TX).

## Results

### Study population characteristics

Of the 157,001 participants, the mean age was 47.0 ± 0.1 years, and balanced in terms of sex/gender (51.1%_women_ vs. 48.9%_men_) (Table 1). Most participants identified as NH-White (68.4%) followed by NH-Black (11.2%), Hispanic/Latinx (14.7%), and NH-Asian (5.7%). Approximately 32.4% of participants earned a college degree, 43.2% had an annual household income ≥ $75,000, 61.1% were married/living with a partner or cohabitating, 37.0% resided in the Southern region of the US, 84.3% never smoked or quit smoking > 12 months prior to baseline, and 66.7% formerly consumed alcohol (> 12 months prior to baseline). Additionally, most lived in households with high food security (83.9%) followed by marginal (6.5%), low (5.3%) and very low (4.3%) (Table 1).

**Table 1:** Age-standardized sociodemographic, health behavior, and clinical characteristics, overall and by household food security status, National Health Interview Survey, 2014–2018, 2020, (*N* = 157,001) ^a^

Characteristics	Household Food Security Status ^h^				
	**Very Low *n* = 6,691 (4.3%)**	**Low *n* = 8,260 (5.3%)**	**Marginal *n* = 10,211 (6.5%)**	**High *n* = 131,839 (83.9%)**	**Overall *N* = 157,001 (100%)**
**Sociodemographic**					
Age, mean ± SE (years)	43.7 ± 0.3	43.6 ± 0.2	43.1 ± 0.2	47.7 ± 0.1	47.0 ± 0.1
18–30	26.2	27.9	29.5	22.1	23.0
31–49	35.4	35.0	35.5	31.8	32.4
≥ 50	38.3	37.1	34.9	46.1	44.6
Sex/gender					
Men	43.2	43.6	43.3	49.9	48.9
Women	56.8	56.4	56.7	50.1	51.1
Race/ethnicity					
NH-White	53.9	47.0	52.4	71.3	68.4
NH-Black	24.0	23.3	19.2	9.4	11.2
Hispanic/Latinx	19.6	26.0	23.7	13.3	14.7
NH-Asian	2.5	3.7	4.7	6.0	5.7
Educational Attainment					
< High School	24.0	24.3	20.2	7.9	10.0
High School graduate	35.3	37.0	36.2	25.8	27.5
Some College	31.9	28.3	29.3	30.2	30.2
≥ College	8.8	10.4	14.3	36.1	32.4
Annual household income					
< $35,000	73.9	67.0	53.8	21.4	27.6
$35-$74,999	21.2	25.4	30.8	29.3	29.1
≥ $75,000	4.9	7.6	15.3	49.2	43.2
Unemployed/not in labor force	64.3	58.5	51.4	37.2	40.3
Marital status					
Divorced/widowed	38.4	33.1	28.4	18.8	20.7
Single/no live-in partner	25.4	23.0	21.1	17.4	18.2
Married/living with partner/co-habited	36.2	43.9	50.5	63.8	61.1
Region of residence					
Northeast	15.2	16.2	17.1	18.1	17.8
Midwest	21.4	18.4	21.2	22.6	22.3
South	43.4	45.2	40.7	36.0	37.0
West	20.0	20.2	21.0	23.3	22.9
**Health Behaviors**					
Smoking status					
Never/quit > 12 months prior	62.1	73.4	76.0	86.6	84.3
Former (quit ≤ 12 months ago)	2.3	1.5	2.0	1.2	1.3
Current	35.6	25.1	22.0	12.2	14.4
Alcohol consumption					
Lifetime abstinence (< 12 drinks in life)	22.1	25.2	21.8	16.7	17.5
Former (no drinks past year)	52.7	52.0	56.2	68.8	66.7
Current (≥ 1 drink past year)	25.3	22.8	22.1	14.5	15.8
Leisure-time physical activity (PA)					
Never/unable	50.6	46.7	42.7	28.6	31.1
Does not meet PA guidelines	18.8	19.7	20.0	19.8	19.8
Meets PA guidelines ^b^	30.6	33.6	37.3	51.7	49.1
Usual sleep duration					
< 6 h	24.2	16.7	13.4	7.7	9.1
< 7 h	49.8	41.3	38.1	29.3	31.2
7–9 h (recommended)	43.1	52.9	56.1	67.2	64.9
> 9 h	7.0	5.8	5.8	3.5	3.9
**Clinical Characteristics**					
Health status					
Excellent/very good/good	53.8	62.5	72.5	89.2	85.7
Fair/poor	46.2	37.5	27.5	10.8	14.3
Body Mass Index (BMI) ^c^					
Underweight (< 18.5 kg/m^2^)	2.1	1.9	1.5	1.6	1.6
Recommended (18.5- < 25 kg/m^2^)	26.9	25.5	25.8	32.6	31.6
Overweight (25–29.9 kg/m^2^)	29.0	30.0	32.8	36.0	35.2
Obesity (> 30 kg/m^2^)	42.1	42.6	39.9	29.9	31.6
Dyslipidemia ^d^	77.1	73.8	67.7	68.2	68.9
Hypertension ^e^	50.6	47.0	41.8	34.3	36.0
Diabetes or type 2 diabetes ^f^	31.3	29.4	24.1	16.9	18.4
Modified ideal cardiovascular health ^g^	1.8	3.3	3.9	9.4	8.4

*Abbreviation: SE* Standard error

^a^Note all estimates are weighted for the survey’s complex sampling design. All estimates are age-standardized to the US 2010 population, except for age. Percentage may not sum to 100 due to missing values or rounding

^b^Meets PA guidelines for Americans, defined as ≥ 150 min/week of moderate intensity or ≥ 75 min/week of vigorous intensity or ≥ 150 min/week of moderate and vigorous intensity

^c^Self-reported weight and height were used to calculate (weight [kg] / height [m^2^]) body mass index

^d^Dyslipidemia defined as currently taking prescribed medicine to lower cholesterol high cholesterol in the 12 months prior to interview

^e^Hypertension defined as ever told on two or more different visits that you have hypertension or high blood pressure or currently taking prescribed medicine to lower blood pressure

^f^Prediabetes defined as ever told by a doctor had prediabetic condition, prediabetes, or borderline diabetes. Type 2 diabetes defined as ever told by a doctor or health professional that you have diabetes or sugar diabetes and being told you have type 2 diabetes

^g^Modified ideal cardiovascular health includes a dichotomized (yes [7]/no [< 7]) summary score for never smoking/quit > 12 months prior to interview, BMI 18.5—< 25 kg/m^2^, meeting physical activity guidelines for Americans, sleep duration of 7–9 h, and no dyslipidemia, hypertension, or prediabetes/type 2 diabetes

^h^Household food security status was captured using the U.S. Department of Agriculture’s U.S. Household Food Security Survey Module 10-item screener. Responses were summed (0–10) and categorized as very low (6–10), low (3–5), marginal (1–2), and high (0) food security

**Fig. 1:**
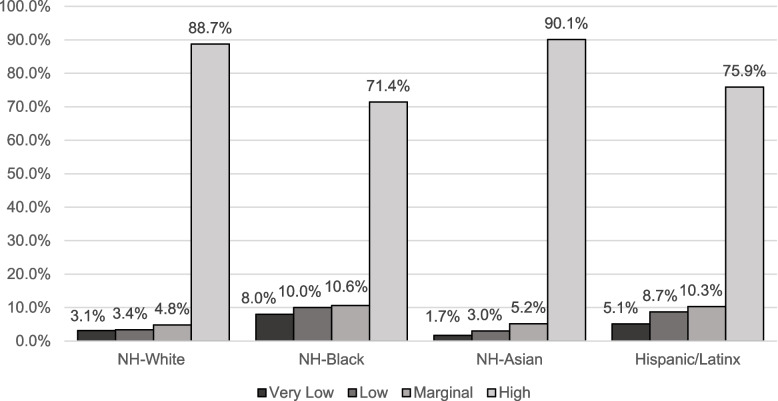
Age-standardized household food security status ^a^ by race/ethnicity, National Health Interview Survey, 2014–2018, 2020, (*N* = 157,001) ^b^. ^a^ Household food security status was captured using the U.S. Department of Agriculture’s U.S. Household Food Security Survey Module 10-item screener. Responses were summed (0–10) and categorized as very low (6–10), low (3–5), marginal (1–2), and high (0) food security. ^b^ Note all estimates are weighted for the survey’s complex sampling design. All estimates are age-standardized to the US 2010 population, except for age. Percentage may not sum to 100 due to missing values or rounding

**Fig. 2:**
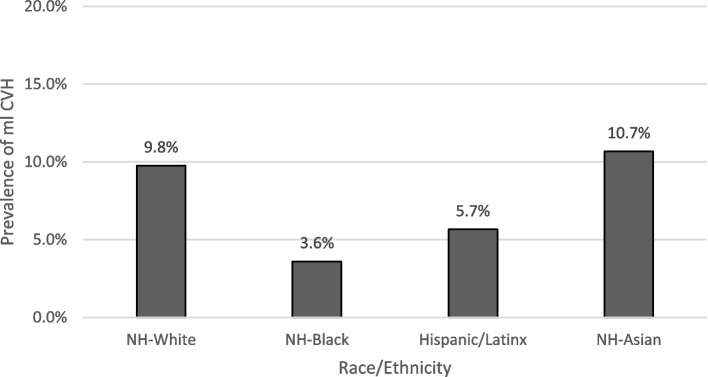
Age-standardized modified ideal cardiovascular health ^a^ by race/ethnicity, National Health Interview Survey, 2014–2018, 2020, (*N* = 157,001) ^b^. Abbreviations: mICVH = Modified ideal cardiovascular health. ^a^ Modified ideal cardiovascular health includes a dichotomized (yes [7]/no [< 7]) summary score for never smoking/quit > 12 months prior to interview, BMI 18.5—< 25 kg/m^2^, meeting physical activity guidelines for Americans, sleep duration of 7–9 h, and no dyslipidemia, hypertension, or prediabetes/type 2 diabetes. ^b^ Note all estimates are weighted for the survey’s complex sampling design. All estimates are age-standardized to the US 2010 population, except for age. Percentage may not sum to 100 due to missing values or rounding

Women versus men had a higher prevalence of very low food security among those who were Hispanic/Latinx (5.5% vs. 4.7%), NH-Asian (1.9% vs. 1.4%), NH-Black (8.6% vs. 7.2%) and NH-White (3.4% vs. 2.7%) (Supplemental Table 2). Additionally, more NH-Black (8.6%) and Hispanic/Latinx (5.5%) women had a higher prevalence of very low food security compared to NH-Asian (1.9%) and NH-White (3.4%) women (Supplemental Table 2). Further, more Hispanic/Latinx (5.1%) and NH-Black (8.0%) adults had a higher prevalence of very low food security compared to NH-Asian (1.7%) and NH-White (3.1%) adults (Fig. 1). Compared to men, women had a higher prevalence of mICVH overall (10.2% vs. 6.7%) (Supplemental Table 3). Similarly, women versus men had a higher mICVH prevalence among those who were NH-White (12.2% vs. 7.3%), Hispanic/Latinx (6.8% vs. 4.5%) and NH-Asian (11.6% vs. 9.6%). However, among NH-Black participants, women had a lower prevalence of mICVH (3.0%) compared to men (4.3%). Further, NH-Asian (10.7%) and NH-White (9.8%) adults had a higher prevalence of mICVH compared to Hispanic/Latinx (5.7%) and NH-Black (3.6%) adults (Fig. 2).

### Food security status and modified ideal cardiovascular health overall

Compared to participants with high food security status, those with very low (PR = 0.34 [95% CI: 0.27–0.43]), low (PR = 0.62 [95% CI: 0.52–0.73]), and marginal (PR = 0.61 [95% CI: 0.54–0.70]) food security had lower mICVH prevalence (Table 2).

**Table 2:** Prevalence ratios of modified ideal cardiovascular health among participants with very low, low, or marginal compared to high food security overall, by sex/gender ^b^, and by race/ethnicity ^c^, National Health Interview Survey, 2014–2018, 2020, (*N* = 157,001)

	**Prevalence Ratio (95% Confidence Interval) for Modified Ideal CVH** ^**a**^** by Household Food Security Status **^**d**^		
	**Very low (*n* = 6,691) vs. High (*n* = 131,839)**	**Low (*n* = 8,260) vs. High (*n* = 131,839)**	**Marginal (*n* = 10,211) vs. High (*n* = 131,839)**
**Overall** (*N* = 157,001)	**0.34**	**0.62**	**0.61**
	**[0.27—0.43]**	**[0.52—0.73]**	**[0.54—0.70]**
Men (*n* = 72,436)	**0.48**	**0.66**	**0.71**
	**[0.35—0.66]**	**[0.51—0.85]**	**[0.58—0.89]**
Women (*n* = 84,565)	**0.23**	**0.59**	**0.56**
	**[0.17—0.31]**	**[0.47—0.75]**	**[0.48—0.66]**
**Hispanic/Latinx** (*n* = 22,022)	**0.41**	**0.70**	0.83
	**[0.27—0.65]**	**[0.52—0.95]**	[0.63—1.09]
Men (*n* = 9904)	**0.53**	0.93	0.89
	**[0.29—0.98]**	[0.62—1.39]	[0.57—1.38]
Women (*n* = 12,118)	**0.29**	**0.55**	0.80
	**[0.15—0.56]**	**[0.36—0.84]**	[0.58—1.11]
**NH-Asian** (*n* = 8458)	**0.36**	0.45	0.73
	**[0.14—0.91]**	[0.18—1.11]	[0.49—1.10]
Men (*n* = 3973)	0.37	0.43	0.73
	[0.08—1.65]	[0.13—1.45]	[0.38—1.39]
Women (*n* = 4,485)	0.35	0.46	0.75
	[0.11—1.18]	[0.13—1.67]	[0.44—1.27]
**NH-Black** (*n* = 18,223)	**0.52**	0.88	0.84
	**[0.30—0.92]**	[0.59—1.31]	[0.57—1.23]
Men (*n* = 7365)	0.58	0.75	0.83
	[0.29—1.18]	[0.43—1.32]	[0.47—1.46]
Women (*n* = 10,858)	**0.34**	0.98	0.83
	**[0.15—0.78]**	[0.55—1.74]	[0.52—1.34]
**NH-White** (*n* = 108,298)	**0.27**	**0.51**	**0.47**
	**[0.19—0.36]**	**[0.40—0.66]**	**[0.39—0.56]**
Men (*n* = 51,194)	**0.40**	**0.44**	**0.57**
	**[0.25—0.63]**	**[0.30—0.66]**	**[0.43—0.76]**
Women (*n* = 57,104)	**0.19**	**0.57**	**0.42**
	**[0.12—0.28]**	**[0.41—0.78]**	**[0.34—0.53]**

Models are adjusted for age (18–30 years, 31–49 years, ≥ 50 years), sex/gender (man, woman), annual household income (< $35,000; $35,000-$74,999; ≥ $75,000), marital status (married/cohabitating, single/no live-in partner, divorced/separated/widowed), educational attainment (< high school, high school graduate, some college, ≥ college), region of residence (Northeast, Midwest, South, West), alcohol consumption (current [heavy], current [≤ moderate], former, lifetime abstainer), and survey year. Models in the total/overall sample are additionally adjusted for race and ethnicity (Hispanic/Latinx, NH-Asian, NH-Black/African American, NH-White)

All estimates are weighted for the complex survey design. Bolded values indicate statistical significance at a two-sided *p*-value < 0.05

*Abbreviations: CVH* Cardiovascular health, *NH* Non-Hispanic, *NE* Not able to estimate

^a^Modified ideal cardiovascular health includes a dichotomized (yes [7]/no [< 7]) summary score for never smoking/quit > 12 months prior to interview, BMI 18.5—< 25 kg/m^2^, meeting physical activity guidelines for Americans, sleep duration of 7–9 h, and no dyslipidemia, hypertension, or prediabetes/type 2 diabetes

^b^Significant interaction effect between sex/gender and food security status on modified ideal CVH (*p* < .0001)

^c^Significant interaction effect between race/ethnicity and food security status on modified ideal CVH (*p* < .0001)

^d^Household food security status was captured using the U.S. Department of Agriculture’s U.S. Household Food Security Survey Module 10-item screener. Responses were summed (0–10) and categorized as very low (6–10), low (3–5), marginal (1–2), and high (0) food security

### Food security status and modified ideal cardiovascular health by sex/gender

The association between very low versus high food security and mICVH was stronger among women (PR = 0.23 [95% CI: 0.17–0.31]) compared to men (PR = 0.48 [95% CI: 0.35–0.66]); p-interaction < 0.01) (Table 2). Women with low (PR = 0.59 [95% CI: 0.47–0.75]), and marginal (PR = 0.56 [95% CI: 0.48–0.66]) versus high food security status had a lower prevalence of mICVH. Men with low, and marginal versus high food security status was associated with a lower prevalence of mICVH; (PR = 0.66 [95% CI: 0.51–0.85]) and (PR = 0.71 [95% CI: 0.58–0.89]) respectively.

### Food security status and modified ideal cardiovascular health by race/ethnicity

Among NH-Black participants, very low versus high food security status was more strongly associated with lower mICVH prevalence (PR = 0.52 [95% CI: 0.30–0.92]; p-interaction < 0.01) compared to those with low or marginal versus high food security status and mICVH prevalence; ((PR = 0.88 [95% CI: 0.59–1.31]) and (PR = 0.84 [95% CI: 0.57–1.23]), respectively). Additionally, very low, low, and marginal versus high food security was associated with lower mICVH prevalence among NH-White participants; ((PR = 0.27 [95% CI: 0.19–0.36]), (PR = 0.51 [95% CI: 0.40–0.66]), and (PR = 0.47 [95% CI: 0.39–0.56]), respectively).

### Food security status and modified ideal cardiovascular health by sex/gender and race/ethnicity

Among Hispanic/Latinx adults, very low versus high food security status was associated with lower mICVH but was stronger among women (PR = 0.29 [95% CI: 0.15–0.56]) compared to men (PR = 0.53 [95% CI: 0.29–0.98]). Compared to those with high food security, low food security status was associated with lower mICVH prevalence (PR = 0.34 [95% CI: 0.15–0.78]) among NH-Black women. Additionally, the association between very low versus high food security among NH-White adults was stronger among women (PR = 0.19 [95% CI: 0.12–0.28]) compared to men (PR = 0.40 [95% CI: 0.25–0.63]).

### Food security status and modified ideal cardiovascular health among minoritized racial/ethnic groups compared to NH-White participants with high food security

Hispanic/Latinx participants with very low, low, marginal, and high food security versus NH-White participants with high food security had lower mICVH prevalence; (PR_very low_ = 0.30 [95% CI: 0.20–0.47]), (PR_low_ = 0.52 [95% CI: 0.38–0.70]), (PR_marginal_ = 0.61 [95% CI: 0.47–0.78]), and (PR_high_ = 0.70 [95% CI: 0.64–0.77]) respectively (Table 3). Compared to NH-White participants with high food security, associations between very low and low food security and lower mICVH prevalence were stronger for Hispanic/Latinx women ((PR_very low_ = 0.22 [95% CI: 0.11–0.42]) and (PR_low_ = 0.42 [95% CI: 0.27–0.64])) versus men ((PR_very low_ = 0.38 [95% CI: 0.21–0.69]) and (PR_low_ = 0.69 [95% CI: 0.46–1.04])). NH-Asian participants with very low or high food security versus NH-White participants with high food security was associated with lower mICVH prevalence; (PR_very low_ = 0.36 [95% CI: 0.14–0.96]) and (PR_high_ = 0.88 [95% CI: 0.79–0.97]) respectively. NH-Black participants with very low, low, marginal, and high food security versus NH-White participants with high food security had lower mICVH prevalence; (PR_very low_ = 0.25 [95% CI: 0.14–0.44]), (PR_low_ = 0.42 [95% CI: 0.29–0.61]), (PR_marginal_ = 0.38 [95% CI: 0.26–0.55]), and (PR_high_ = 0.41 [95% CI: 0.36–0.47]) respectively. In terms of differences by sex/gender and race/ethnicity, associations between very low security compared to NH-White participants with high food security and lower mICVH prevalence were significantly stronger for NH-Black women (PR_very low_ = 0.11 [95% CI: 0.05–0.24]) and versus NH-Black men (PR_very low_ = 0.50 [95% CI: 0.26–0.93]).

**Table 3:** Prevalence ratios of modified ideal cardiovascular health among Hispanic/Latinx, non-Hispanic Asian, and non-Hispanic Black participants with very low, low, marginal, and high food security compared to non-Hispanic White participants with high food security, National Health Interview Survey, 2014–2018, 2020, (*N* = 144,019)

	**Prevalence Ratio (95% Confidence Interval) for Modified Ideal CVH** ^**a**^ **by Household Food Security Status **^**b**^			
	**Very low (*n* = 3,036) vs. NH-White High (*n* = 95,316)**	**Low (*n* = 4,288) vs. NH-White High (*n* = 95,316)**	**Marginal (*n* = 4,856) vs. NH-White High (*n* = 95,316)**	**High (*n* = 36,523) vs. NH-White High (*n* = 95,316)**
**Overall** (*N* = 144,019)	**0.28**	**0.47**	**0.53**	**0.65**
	**[0.20—0.39]**	**[0.37—0.58]**	**[0.43—0.64]**	**[0.61—0.69]**
Men (*n* = 66,982)	**0.43**	**0.64**	**0.67**	**0.74**
	**[0.28—0.66]**	**[0.47—0.88]**	**[0.50—0.90]**	**[0.67—0.82]**
Women (*n* = 77,037)	**0.17**	**0.36**	**0.46**	**0.60**
	**[0.11—0.27]**	**[0.26—0.50]**	**[0.36—0.58]**	**[0.55—0.65]**
**Hispanic/Latinx** (*n* = 22,022)	**0.30**	**0.52**	**0.61**	**0.70**
	**[0.20—0.47]**	**[0.38—0.70]**	**[0.47—0.78]**	**[0.64—0.77]**
Men (*n* = 9,904)	**0.38**	0.69	**0.65**	**0.70**
	**[0.21—0.69]**	[0.46—1.04]	**[0.43—0.99]**	**[0.61—0.80]**
Women (*n* = 12,118)	**0.22**	**0.42**	**0.59**	**0.72**
	**[0.11—0.42]**	**[0.27—0.64]**	**[0.43—0.82]**	**[0.64—0.80]**
**NH-Asian** (*n* = 8,458)	**0.36**	0.44	0.77	**0.88**
	**[0.14—0.96]**	[0.17—1.13]	[0.52—1.15]	**[0.79—0.97]**
Men (*n* = 3,973)	0.30	0.44	0.80	0.99
	[0.06—1.41]	[0.13—1.46]	[0.42—1.52]	[0.86—1.15]
Women (*n* = 4,485)	0.44	0.45	0.78	**0.81**
	[0.13—1.48]	[0.12—1.72]	[0.46—1.32]	**[0.7—0.92]**
**NH-Black** (*n* = 18,223)	**0.25**	**0.42**	**0.38**	**0.41**
	**[0.14—0.44]**	**[0.29—0.61]**	**[0.26—0.55]**	**[0.36—0.47]**
Men (*n* = 7,365)	**0.50**	0.63	0.67	**0.62**
	**[0.26—0.93]**	[0.37—1.08]	[0.39—1.17]	**[0.51—0.75]**
Women (*n* = 10,858)	**0.11**	**0.31**	**0.25**	**0.30**
	**[0.05—0.24]**	**[0.18—0.53]**	**[0.16—0.39]**	**[0.25—0.35]**

Models are adjusted for age (18–30 years, 31–49 years, ≥ 50 years), gender (man, woman), annual household income (< $35,000, $35,000-$74,999, ≥ $75,000), marital status (married/cohabitating, single/no live-in partner, divorced/separated/widowed), educational attainment (< high school, high school graduate, some college, ≥ college), region of residence (Northeast, Midwest, South, West), alcohol consumption (current [heavy], current [≤ moderate], former, lifetime abstainer), and survey year. Models in the total/overall sample are additionally adjusted for race and ethnicity (Hispanic/Latinx, NH-Asian, NH-Black/African American, NH-White)

All estimates are weighted for the complex survey design. Bolded values indicate statistical significance at a two-sided *p*-value < 0.05

*Abbreviations: CVH* Cardiovascular health, *NH* Non-Hispanic, *NE* Not able to estimate

^a^Modified ideal cardiovascular health includes a dichotomized (yes [7]/no [< 7]) summary score for never smoking/quit > 12 months prior to interview, BMI 18.5—< 25 kg/m^2^, meeting physical activity guidelines for Americans, sleep duration of 7–9 h, and no dyslipidemia, hypertension, or prediabetes/type 2 diabetes

^b^Household food security status was captured using the U.S. Department of Agriculture’s U.S. Household Food Security Survey Module 10-item screener. Responses were summed (0–10) and categorized as very low (6–10), low (3–5), marginal (1–2), and high (0) food security

## Discussion

In this nationally representative and racially/ethnically diverse study, we investigated household food security status in relation to mICVH prevalence among US adults. Very low, low, and marginal compared to high food security status was associated with lower mICVH prevalence. These findings aligned with our hypothesis. Contrary to our hypothesis, stronger associations between lower food security status and lower mICVH prevalence were observed among NH-White adults compared to associations within racially/ethnically minoritized groups. However, the burden of low and very low food security prevalence was higher among racially/ethnically minoritized groups in comparison to NH-White adults. Additionally, lower mICVH was observed among minoritized racial/ethnic groups when comparing Hispanic/Latinx, NH-Asian, and NH-Black adults with high food security compared to NH-White adults with high food security. Stronger statistically significant associations between very low, low, and marginal versus high food security in relation to lower mICVH prevalence were observed among women compared to men.

We found that NH-Black and Hispanic/Latinx adults had higher levels of very low and low food security status compared to NH-White and NH-Asian adults. Prior literature has also described racial/ethnic differences in distributions of both food security status and mICVH prevalence, with racially/ethnically minoritized groups being less likely to be food secure or have mICVH than NH-White adults [1, 26, 31, 32]. Considering that our study observed racial/ethnic disparities in mICVH prevalence, even among racially/ethnically minoritized groups with high food security, additional social determinants (e.g., interpersonal racial discrimination; job strain) of mICVH disparities may exist beyond food security itself. For example, the American Heart Association recently identified structural racism as a fundamental driver of health disparities in the US [41, 42]. While race is a social construct primarily predicated on phenotype (e.g., skin color; hair texture), structural racism has created and perpetuated differential access to power, resources, and opportunities in ways that advantage NH-White individuals while synchronously disadvantaging minoritized racial and ethnic groups [41–43]. The subsequent social and environmental contextual level factors (e.g., community and individual level stress) stemming from structural racism (e.g., limited neighborhood resources following residential segregation) are hypothesized drivers of racial and ethnic health disparities and may partially explain why our study observed weaker relative associations between lower food security and low mICVH prevalence among adults from minoritized racial/ethnic groups compared to NH-White adults [41–44]. When comparing minoritized racial/ethnic groups with high vs. low prevalence of food insecurity, along with a multitude of other adverse exposures – caused by concentrated and cumulative disadvantage that increase disease risk (i.e., non-ideal CVH) – the impact of a particular adverse exposure (i.e., food insecurity) can be expected to be more difficult to detect. The unexposed group (e.g., without food insecurity) among minoritized groups compared to non-minoritized groups has more risk factors for non-ideal CVH, which can make relative associations for one particular adverse exposure appear weaker in minoritized racial/ethnic groups with more risk factors. Nonetheless, the exposure and outcome burden (based on absolute estimates of prevalence) is higher among minoritized groups, which is more informative for public health impact than relative estimates (most relevant for disease etiology). Ultimately, future research that considers the multifactorial determinants of CVH disparities and its complex manifestations are warranted, but food insecurity is indeed a plausible contributor.

Subgroup analyses in our study yielded differential associations between food insecurity and mICVH after stratifying by sex/gender and race/ethnicity. For example, very low versus high food security was associated with lower mICVH for each racial/ethnic group, but strongest among women who were NH-White. However, when comparing racially/ethnically minoritized groups with very low food security to NH-White adults with high food security and lower mICVH, associations were the strongest for NH-Black women. Those findings align with one study that found that women compared to men living in food insecure households were more likely to be NH-Black, irrespective of advanced educational attainment [5]. Additionally, some evidentiary support in the literature also suggests that food insecure households are most likely to be headed by women versus men and NH-Black or Hispanic/Latinx versus NH-White adults [1, 4, 5]. While food insecurity was found to be associated with lower mICVH overall, and when comparing racially/ethnically minoritized to NH-White adults, its deleterious effect may be stronger among Hispanic/Latinx and NH-Back compared to NH-White women [45]. Considering the interconnectedness of systems of power (e.g., race, gender, socioeconomic status) shaping oppression, as well as privilege, additional studies incorporating an intersectional framework approach to investigate food insecurity and CVH inequities are warranted [34]. Such studies are vital for elucidating mechanistic pathways driving inequities in food insecurity and poor CVH observed among groups with multiple identities enduring oppression (e.g., women from minoritized racial/ethnic groups living below the poverty line).

Although many public health strategies in the US aimed at reducing mICVH disparities have historically focused more on downstream, individual level factors (e.g., health behaviors), addressing upstream, community level factors (e.g., the neighborhood food environment) may be equally as important. Policies aimed at addressing upstream drivers of social determinants can help alleviate the burden of food insecurity, which may otherwise promote and exacerbate health inequities that are systemically linked (e.g., food insecurity, obesity and type 2 diabetes) [21, 46, 47]. However, it will be important to delineate components of upstream community level factors, including how they may contribute towards or reduce food insecurity. For example, food apartheid in the US has subsequently contributed towards limited access to affordable healthy food options in neighborhoods that are predominantly comprised of racial/ethnic minoritized groups who live in concentrated poverty [13–17]. Further, limited availability of affordable healthy food in those neighborhoods potentially increase cumulative risk for mICVH disparities, which may be compounded by additional social determinants [10, 11, 13, 14, 16, 48]. In fact, one study found that food swamps (with calorie dense, nutrient poor products) rather than ‘food deserts’ may be a stronger indicator of obesity risk [49]. While addressing food apartheid will be necessary, policies aimed at eliminating food swamps and providing access to healthy options (e.g., fruits; vegetables) should be prioritized to reduce food insecurity disparities. Policies that help grant access (e.g., food trucks with produce) and reduce the cost of healthier food options in low-income communities may be a way to afford and ultimately purchase healthy food options that may exist in their neighborhoods but otherwise are unaffordable. Additionally, future policies seeking to take a harm reduction (strategies that seek to minimize the unfavorable effects of health behaviors) approach should focus on limiting serving sizes of energy-dense, low nutrient items in a non-punitive way [50]. Such approaches may be promising for combating food insecurity disparities among racially/ethnically minoritized groups, despite cohabitating with food swamps.

Our study has limitations. First, we relied on a cross-sectional study design, which precludes causal interpretations. Additionally, the average response rate was lower than in previous years (potentially due to the 2020 survey year occurring during the COVID-19 pandemic). If the lower average response rate in the 2020 survey is attributed to the COVID-19 pandemic, food insecurity may be underreported, which potentially produced underestimates of associations between food security status and mICVH reported in this study. Further, we excluded 2019 NHIS data in our study, as data on sleep duration were not collected during the 2019 survey year. It is worth noting that the omission of the 2019 survey year, due to missing data on sleep, may have reduced the power in our study due to a smaller sample size. Next, food security and mICVH measures (for which the updated mICVH metric inclusive of sleep has not yet been validated) were self-reported, which may have resulted in underreporting (e.g., due to social desirability bias, not recently having a physical examination, etc.) and thus may have resulted in the underestimation of associations between food security study and mICVH. Further, because the addition of sleep to the mICVH metric was not validated, misclassification of mICVH may vary by race/ethnicity due to inequities in high quality healthcare access [51]. Therefore, future research using objective and validated measures is warranted. However, the addition of sleep as an mICVH metric contributes to the novelty of our study. Data on diet were not collected by the NHIS, which is why we did not model our measure based on the AHA’s Life’s Essential 8 metric, which includes an assessment of diet. While underrepresented populations (e.g., gender minority and other understudied racial/ethnic groups) were not included in this study, our observation that ‘very low’, ‘low’, and ‘marginal’ versus ‘high’ household food security status—which disproportionally burdens minoritized racial/ethnic adults (particularly women)—was associated with lower mICVH prevalence, underscoring the need to address inequities in food security to reduce racial/ethnic CVH disparities [1, 3–6, 26, 31].

Despite these limitations, our study also has noteworthy strengths that extend the scientific literature. For instance, we used a large, nationally representative, and racially/ethnically diverse sample of the US, which allowed us to examine the intersectionality of sex/gender and race/ethnicity. This ‘National Health Interview Survey’ data source is used to monitor the health of the nation. To our knowledge, we are among the first study to investigate associations between food insecurity and ICVH, inclusive of the newly added sleep metric, using a nationally representative US sample. Furthermore, the USDA’s U.S. Household Food Security Survey Module scale used to collect data on food security has been previously validated [52]. Future large, longitudinal studies (that oversample racially/ethnically minoritized and other historically excluded individuals) investigating drivers of ICVH and food security disparities are needed.

## Conclusions

We found that ‘very low,’ ‘low.’ and ‘marginal’ compared to ‘high’ food security status was associated with lower mICVH prevalence, using the updated mICVH metric that now includes sleep duration. Stronger magnitude of associations between very low, low and marginal versus high food security and lower mICVH were observed when comparing women to men. Additionally, lower mICVH was observed among minoritized racial/ethnic groups when comparing racially/ethnically minoritized adults with high food security to NH-White adults with high food security. This finding underscores the need to prioritize initiatives directed at meeting basic needs by achieving food security equity to prevent further exacerbating mICVH disparities, particularly among already identified socially vulnerable populations. Addressing upstream drivers of social determinants (e.g., financial strain; food apartheid) can help alleviate the burden of food insecurity, which may exacerbate the health inequities projected to widen due to the worsening climate crisis [21, 46, 47]. Ultimately, developing and enforcing effective and sustainable policies, programs, and practices that address upstream drivers of food insecurity in the US are imperative for promoting CVH equity.

### Supplementary Information

**Supplementary Material 1.**

**Supplementary Material 2.**

**Supplementary Material 3.**

**Supplementary Material 4.**

## Data Availability

The datasets analyzed during the current study are from the National Health Interview Survey, which is publicly available and was retrieved from https://www.cdc.gov/nchs/nhis/index.htm.

## Abbreviations

AHA: American Heart Association
BMI: Body Mass Index
CI: Confidence Interval
CVD: Cardiovascular Disease
CVH: Cardiovascular Health
mICVH: Modified Ideal Cardiovascular Health
MCC: Multiple Chronic Conditions
METs: Metabolic Equivalents
NH: Non-Hispanic
NHIS: National Health Interview Survey
PR: Prevalence Ratio
SES: Socioeconomic Status
USDA: United States Department of Agriculture
USD: United States Dollars

## References

[1] Coleman-Jensen A, Gregory CA, Singh A. Household Food Security in the United States in 2013 (September 1, 2014). USDAERS Economic Research Report Number 173. 2014. Available at SSRN: 10.2139/ssrn.2504067.

[2] Benjamin EJ, Muntner P, Alonso A (2019). Heart disease and stroke statistics—2019 update: a report from the American Heart Association. Circulation.

[3] Coleman-Jensen A, Rabbitt MA, Gregory CA, Singh A. Household Food Security in the United States in 2020, ERR-298, U.S. Department of Agriculture. Econ Res Serv. 2021.

[4] Jung NM, de Bairros FS, Pattussi MP, Pauli S, Neutzling MB (2017). Gender differences in the prevalence of household food insecurity: a systematic review and meta-analysis. Public Health Nutr.

[5] Ma C, Ho SK, Singh S, Choi MY (2021). Gender disparities in food security, dietary intake, and nutritional health in the United States. Am Coll Gastroenterol.

[6] Hernandez DC, Reesor LM, Murillo R (2017). Food insecurity and adult overweight/obesity: gender and race/ethnic disparities. Appetite.

[7] Clay LA, Slotter R, Heath B, Lange V, Colón-Ramos U (2021). Capturing disruptions to food availability after disasters: assessing the food environment following Hurricanes florence and María. Disaster Med Pub Health Prep..

[8] Patz JA, Epstein PR, Burke TA, Balbus JM (1996). Global climate change and emerging infectious diseases. JAMA.

[9] Willett W, Rockström J, Loken B (2019). Food in the Anthropocene: the EAT–Lancet Commission on healthy diets from sustainable food systems. The lancet.

[10] Braveman P, Gottlieb L (2014). The social determinants of health: it’s time to consider the causes of the causes. Public Health Rep.

[11] Braveman P, Egerter S, Williams DR (2011). The social determinants of health: coming of age. Annu Rev Public Health.

[12] Powell-Wiley TM, Baumer Y, Baah FO (2022). Social determinants of cardiovascular disease. Circ Res.

[13] Crowe J, Lacy C, Columbus Y (2018). Barriers to food security and community stress in an urban food desert. Urban Science.

[14] Shaker Y, Grineski SE, Collins TW, Flores AB (2022). Redlining, racism and food access in US urban cores. Agriculture and Human Values..

[15] Bower KM, Thorpe RJ, Rohde C, Gaskin DJ (2014). The intersection of neighborhood racial segregation, poverty, and urbanicity and its impact on food store availability in the United States. Prev Med.

[16] Ekenga CC, Tian R (2022). Promoting food equity in the context of residential segregation. Environ Justice.

[17] Zhang M, Ghosh D (2016). Spatial supermarket redlining and neighborhood vulnerability: a case study of H artford, C onnecticut. Trans GIS.

[18] De Master KT, Daniels J (2019). Desert wonderings: reimagining food access mapping. Agric Hum Values.

[19] Brones A. Karen Washington: It’s not a food desert, it’s food apartheid. Guernica Magazine. 2018;7. https://scholar.google.com/scholar_lookup?title=Karen+Washington:+It%27s+not+a+food+desert,+it%27s+food+apartheid&author=Brones+A&publication_year=2018&pages=7.

[20] Gripper AB, Nethery R, Cowger TL, White M, Kawachi I, Adamkiewicz G (2022). Community solutions to food apartheid: a spatial analysis of community food-growing spaces and neighborhood demographics in Philadelphia. Soc Sci Med.

[21] Strings S, Ranchod YK, Laraia B, Nuru-Jeter A (2016). Race and sex differences in the association between food insecurity and type 2 diabetes. Ethn Dis.

[22] Hall MH, Casement MD, Troxel WM (2015). Chronic stress is prospectively associated with sleep in midlife women: the SWAN sleep study. Sleep.

[23] Liu Y, Njai RS, Greenlund KJ, Chapman DP, Croft JB (2014). Relationships between housing and food insecurity, frequent mental distress, and insufficient sleep among adults in 12 US States, 2009.

[24] Troxel WM, Haas A, Ghosh-Dastidar B (2020). Food insecurity is associated with objectively measured sleep problems. Behav Sleep Med.

[25] Jackson CL, Redline S, Emmons KM (2015). Sleep as a potential fundamental contributor to disparities in cardiovascular health. Annu Rev Public Health.

[26] Lloyd-Jones DM, Allen NB, Anderson CAM (2022). Life’s Essential 8: updating and enhancing the American Heart Association’s construct of cardiovascular health: a presidential advisory from the American Heart Association. Circulation.

[27] Wu S, Huang Z, Yang X (2012). Prevalence of ideal cardiovascular health and its relationship with the 4-year cardiovascular events in a northern Chinese industrial city. Circulation.

[28] Ford ES, Greenlund KJ, Hong Y (2012). Ideal cardiovascular health and mortality from all causes and diseases of the circulatory system among adults in the United States. Circulation.

[29] Machado LBM, Silva BLS, Garcia AP (2018). Ideal cardiovascular health score at the ELSA-Brasil baseline and its association with sociodemographic characteristics. Int J Cardiol.

[30] Simon M, Boutouyrie P, Narayanan K (2017). Sex disparities in ideal cardiovascular health. Heart.

[31] Mujahid MS, Moore LV, Petito LC, Kershaw KN, Watson K, Diez Roux AV (2017). Neighborhoods and racial/ethnic differences in ideal cardiovascular health (the Multi-Ethnic Study of Atherosclerosis). Health Place.

[32] Virani SS, Alonso A, Benjamin EJ (2020). Heart disease and stroke statistics—2020 update: a report from the American Heart Association. Circulation.

[33] Baird R (2008). The impact of climate change on minorities and indigenous peoples.

[34] Bowleg L (2012). The problem with the phrase women and minorities: intersectionality—an important theoretical framework for public health. Am J Public Health.

[35] Moriarity C, Parsons VL, Jonas K, Schar BG, Bose J, Bramlett MD. Sample design and estimation structures for the National Health Interview Survey, 2016–2025; 2022. 35796667

[36] Coleman-Jensen A RM, Hashad RN, Hales L, Gregory CA. US household food security survey module: Three-stage design with screeners. Economic Research Service. Washington DC, USA: US Department of Agriculture; 2012.

[37] Keenan DP, Olson C, Hersey JC, Parmer SM (2001). Measures of food insecurity/security. J Nutr Educ.

[38] Service ER (2012). US adult food security survey module: three stage design, with screeners.

[39] Piercy KL, Troiano RP, Ballard RM (2018). The physical activity guidelines for Americans. JAMA.

[40] Lloyd-Jones DM, Hong Y, Labarthe D (2010). Defining and setting national goals for cardiovascular health promotion and disease reduction: the American Heart Association’s strategic impact goal through 2020 and beyond. Circulation.

[41] Churchwell K, Elkind MS, Benjamin RM (2020). Call to action: structural racism as a fundamental driver of health disparities: a presidential advisory from the American Heart Association. Circulation.

[42] Michos ED, Khan SS (2021). Further understanding of ideal cardiovascular health score metrics and cardiovascular disease. Expert Rev Cardiovasc Ther.

[43] Williams DR (1997). Race and health: basic questions, emerging directions. Ann Epidemiol.

[44] Gee GC, Payne-Sturges DC (2004). Environmental health disparities: a framework integrating psychosocial and environmental concepts. Environ Health Perspect.

[45] Ward JB, Gartner DR, Keyes KM, Fliss MD, McClure ES, Robinson WR (2019). How do we assess a racial disparity in health? Distribution, interaction, and interpretation in epidemiological studies. Ann Epidemiol.

[46] Ashe KM, Lapane KL (2018). Food insecurity and obesity: exploring the role of social support. J Womens Health.

[47] Stuff JE, Casey PH, Connell CL (2007). Household food insecurity and obesity, chronic disease, and chronic disease risk factors. J Hunger Environ Nutr.

[48] McClintock N (2011). From industrial garden to food desert. Cultivating food justice: race, class, and sustainability.

[49] Cooksey-Stowers K, Schwartz MB, Brownell KD (2017). Food swamps predict obesity rates better than food deserts in the United States. Int J Environ Res Public Health.

[50] Johnson KE (2020). Food democracy, health disparities and the New York City trans fat policy. Public Health Nutr.

[51] Fiscella K, Sanders MR (2016). Racial and ethnic disparities in the quality of health care. Annu Rev Public Health.

[52] Blumberg SJ, Bialostosky K, Hamilton WL, Briefel RR (1999). The effectiveness of a short form of the Household Food Security Scale. Am J Public Health.

