# Supplementary material for: Food security status and cardiometabolic health by sex/gender and race/ethnicity among adults in the United States
Source: BMC Public Health. 2024 May 3;24:1220. doi: 10.1186/s12889-024-18655-y (PMC11065684; doi:10.1186/s12889-024-18655-y)
Supplement: Supplementary file 1 — Supplementary Material 1. [file 12889_2024_18655_MOESM1_ESM.docx]

# Supplemental Figure 1: Flow Chart of Study Participants

National Health Interview Survey,

2014-2018, 2020
 (n = 501,332)

Non-Adults (≤17 years old)
(n = 314,208)

Missing Race/Ethnicity
(n = 5,068)

Potentially eligible
 (n = 182,056)

Missing Age
(n = 69)

Missing Gender
(n = 2)

Missing Modified Ideal Cardiovascular Health
(n = 11,430)

Missing Alcohol Use
(n = 935)

Missing Marital Status
(n = 336)

Missing Annual Household Income
(n = 11,889)

Missing Household Food Security Status
(n = 33)

Missing Educational Attainment
(n = 361)

Study Sample
(n = 157,001)
