# Supplementary material for: Food security status and cardiometabolic health by sex/gender and race/ethnicity among adults in the United States
Source: BMC Public Health. 2024 May 3;24:1220. doi: 10.1186/s12889-024-18655-y (PMC11065684; doi:10.1186/s12889-024-18655-y)
Supplement: Supplementary file 3 — Supplementary Material 3. [file 12889_2024_18655_MOESM3_ESM.docx]

# Supplemental Table 2. Age-Standardized Household Food Security Status by Race/Ethnicity and Sex/Gender, National Health Interview Survey, 2014-2018, 2020, (N=157,001) ^a^

|  | **NH-White** | | **NH-Black** | | **Hispanic/Latinx** | | **NH-Asian** | |
| --- | --- | --- | --- | --- | --- | --- | --- | --- |
|  | **Men** | **Women** | **Men** | **Women** | **Men** | **Women** | **Men** | **Women** |
| **Very Low** | 2.7% | 3.4% | 7.2% | 8.6% | 4.7% | 5.5% | 1.4% | 1.9% |
| **Low** | 3.0% | 3.8% | 8.8% | 11.0% | 8.3% | 9.1% | 2.9% | 3.1% |
| **Marginal** | 4.2% | 5.4% | 9.7% | 11.3% | 9.2% | 11.3% | 4.8% | 5.4% |
| **High** | 90.1% | 87.4% | 74.3% | 69.1% | 77.8% | 74.1% | 90.9% | 89.5% |

^a^ Note all estimates are weighted for the survey’s complex sampling design. All estimates are age-standardized to the US 2010 population, except for age. Percentage may not sum to 100 due to missing values or rounding
