# Supplementary material for: Food security status and cardiometabolic health by sex/gender and race/ethnicity among adults in the United States
Source: BMC Public Health. 2024 May 3;24:1220. doi: 10.1186/s12889-024-18655-y (PMC11065684; doi:10.1186/s12889-024-18655-y)
Supplement: Supplementary file 4 — Supplementary Material 4. [file 12889_2024_18655_MOESM4_ESM.docx]

# Supplemental Table 3. Age-Standardized Modified Ideal Cardiovascular Health by Race/Ethnicity and Sex/Gender, National Health Interview Survey, 2014-2018, 2020, (N=157,001) ^a^

| **Overall** | | **NH-White** | | **NH-Black** | | **Hispanic/Latinx** | | **NH-Asian** | |
| --- | --- | --- | --- | --- | --- | --- | --- | --- | --- |
| **Men** | **Women** | **Men** | **Women** | **Men** | **Women** | **Men** | **Women** | **Men** | **Women** |
| 6.7% | 10.2% | 7.3% | 12.2% | 4.3% | 3.0% | 4.5% | 6.8% | 9.6% | 11.6% |

^a^ Note all estimates are weighted for the survey’s complex sampling design. All estimates are age-standardized to the US 2010 population, except for age. Percentage may not sum to 100 due to missing values or rounding
